# Gating of Long-Term Potentiation by Nicotinic Acetylcholine Receptors at the Cerebellum Input Stage

**DOI:** 10.1371/journal.pone.0064828

**Published:** 2013-05-31

**Authors:** Francesca Prestori, Claudia Bonardi, Lisa Mapelli, Paola Lombardo, Rianne Goselink, Maria Egle De Stefano, Daniela Gandolfi, Jonathan Mapelli, Daniel Bertrand, Martijn Schonewille, Chris De Zeeuw, Egidio D’Angelo

**Affiliations:** 1 Brain Connectivity Center, C. Mondino National Institute of Neurology Foundation, IRCCS, Pavia, Italy; 2 Department of Brain and Behavioural Sciences, University of Pavia, Pavia, Italy; 3 Department of Neuroscience, Erasmus MC, Rotterdam, The Netherlands; 4 Pasteur Institute–Cenci Bolognetti Foundation, Department of Biology and Biotechnology “Charles Darwin” Sapienza University of Rome, Rome, Italy; 5 Department of Neurosciences, Medical Faculty, University of Geneva, Geneva, Switzerland; 6 Netherlands Institute for Neuroscience, Royal Dutch Academy of Arts & Sciences (KNAW), Amsterdam, The Netherlands; 7 Department of Biomedical Sciences, University of Modena and Reggio Emilia, Modena, Italy; 8 “Daniel Bovet” Center for Research in Neurobiology, Sapienza University of Rome, Rome, Italy; Neuroscience Campus Amsterdam, VU University, Netherlands

## Abstract

The brain needs mechanisms able to correlate plastic changes with local circuit activity and internal functional states. At the cerebellum input stage, uncontrolled induction of long-term potentiation or depression (LTP or LTD) between mossy fibres and granule cells can saturate synaptic capacity and impair cerebellar functioning, which suggests that neuromodulators are required to gate plasticity processes. Cholinergic systems innervating the cerebellum are thought to enhance procedural learning and memory. Here we show that a specific subtype of acetylcholine receptors, the α7-nAChRs, are distributed both in cerebellar mossy fibre terminals and granule cell dendrites and contribute substantially to synaptic regulation. Selective α7-nAChR activation enhances the postsynaptic calcium increase, allowing weak mossy fibre bursts, which would otherwise cause LTD, to generate robust LTP. The local microperfusion of α7-nAChR agonists could also lead to *in vivo* switching of LTD to LTP following sensory stimulation of the whisker pad. In the cerebellar flocculus, α7-nAChR pharmacological activation impaired vestibulo-ocular-reflex adaptation, probably because LTP was saturated, preventing the fine adjustment of synaptic weights. These results show that gating mechanisms mediated by specific subtypes of nicotinic receptors are required to control the LTD/LTP balance at the mossy fibre-granule cell relay in order to regulate cerebellar plasticity and behavioural adaptation.

## Introduction

Long-term potentiation and depression (LTP and LTD) are forms of synaptic plasticity thought to constitute the biological basis of learning and memory in the brain [1]. In order to effectively regulate behaviour and prevent undesired synaptic modifications, LTP and LTD need to be controlled by local circuit mechanisms and by neuromodulatory systems [2], [3]. LTP and LTD are thought to be distributed in highly specific patterns in neural circuits and indiscriminate induction of these forms plasticity can saturate synaptic capacity, corrupting memory patterns [4]. In the cerebellum, signals entering the granular layer through the mossy fibres can generate LTP and LTD [5]–[7], but the mossy fibre-granule cell relay does not appear to have specific monitoring systems capable of controlling plasticity. Marr originally predicted that LTP at mossy fibre-granule cell synapses, if uncontrolled, would saturate synaptic capacity, impairing cerebellar functioning [6], [8] and consequently this form of plasticity was not considered in subsequent theoretical models [9]. However, it has recently been suggested that gating mechanisms controlled by neuromodulators could hold the key to LTP regulation and LTD induction in the cerebellum granular layer, improving cerebellar adaptive control [3], [10].

Nicotinic acetylcholine receptors (nAChRs) are a family of ligand-gated ion channels abundantly expressed throughout the central nervous system, which can regulate learning and memory in relation to various cognitive processes in animals and humans [11]. Dysfunction of the nAChR system is implicated in nicotine addiction and several brain pathologies, including Alzheimer’s disease, Parkinson’s disease, genetically transmissible epilepsies, ataxia and autism [12]–[14]. In particular, α7-nAChRs are abundantly expressed in the brain at both pre- and postsynaptic localisations [15]–[23] and can be activated by ACh as well as by its metabolite, choline, produced through ACh-esterase-mediated hydrolysis [11]. Since α7-nAChRs are rapidly desensitised [24], they can generate a time-limited cellular signal. In addition, α7-nAChRs are highly calcium permeable (about 10 times more than α4β2-nAChR, the other major receptor subtype in the brain) [15], [25]. Therefore, α7-nAChRs would be capable of dynamically regulating synaptic plasticity.

Whereas nAChRs have been shown to modify long-term synaptic plasticity, often involving α7-nAChR-mediated mechanisms, in the hippocampus, cerebral cortex and striatum [18], [19], [26]–[32], the localisation and impact of these receptors in the cerebellum remains to be elucidated. The present results indicate that α7-nAChR-mediated regulation of the LTP/LTD balance in the granular layer provides an effective mechanism for controlling cerebellar learning.

## Results

### α7-nAChR Activation at the Mossy Fibre-granule Cell Synapse of the Cerebellum

Synaptic transmission at the mossy fibre-granule cell synapse is underpinned by AMPA and NMDA receptors which generate a fast and a slow component of the EPSC, respectively. In the present experiments, unless otherwise stated, we analysed the AMPA current peak, which provides information on the underlying neurotransmission processes [33].

**Figure 1:**
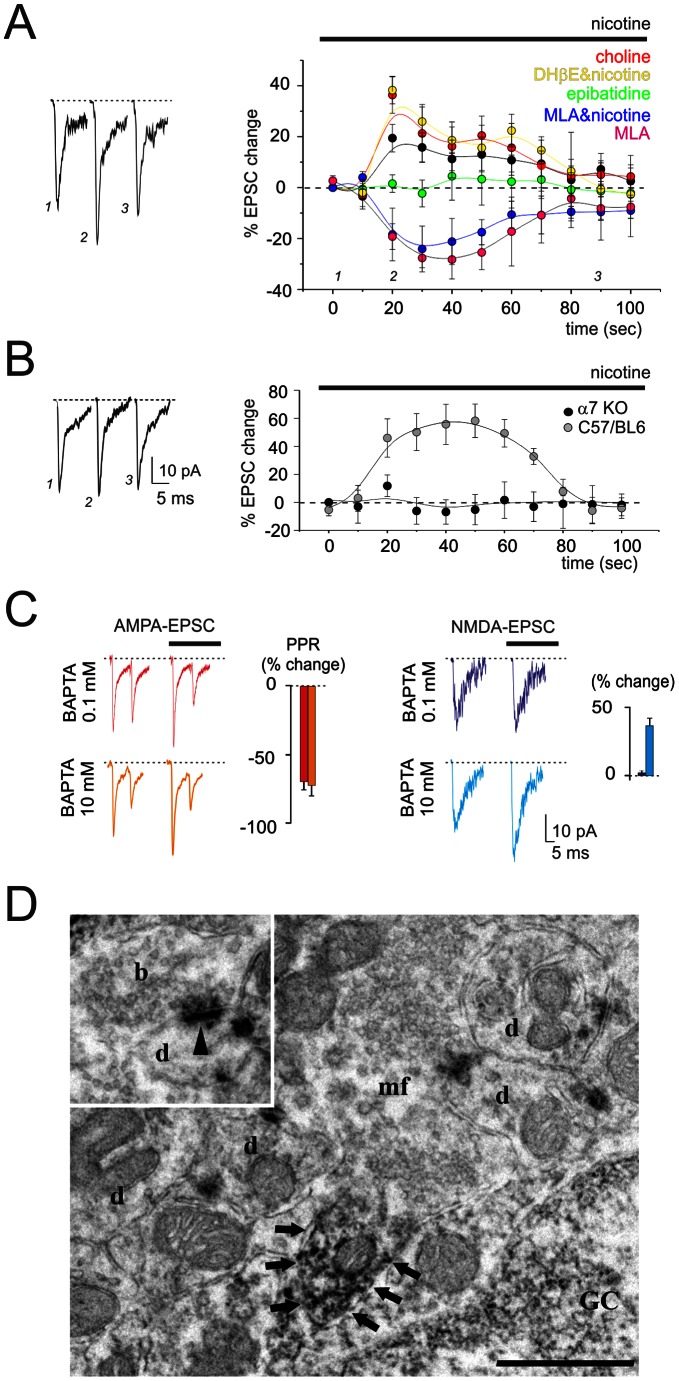
α7-nAChR activation at the mossy fibre-granule cell synapse. In A, B, and C, patch-clamp recordings performed from granule cells voltage-clamped at –70 mV in cerebellar slices. Drugs were applied for 100 seconds (black bar). (**A**) EPSC amplitude changes caused by application of nicotinic agents. (*Left)* Average traces of 10 contiguous EPSCs taken from a representative experiment (1 µM nicotine). *(Right)* Time course of EPSC amplitude changes (mean ± SEM) during application of 1 µM nicotine (n = 56), 10 mM choline (n = 5), 1 µM nicotine +10 nM MLA (n = 4), 50 nM epibatidine (n = 4), 10 nM MLA (n = 5), 1µM DHβE (n = 7). (**B**) EPSC amplitude changes in C57/BL6 (n = 4) and α7-nAChR KO mice (n = 4) during application of 1 µM nicotine. Same panel layout as in A. (**C**) The effect of postsynaptic calcium buffering (0.1 mM or 10 mM intracellular BAPTA) on the action of 1 µM nicotine (black bar). *(Left)* Average traces from 10 contiguous AMPA-EPSCs and NMDA-EPSCs (isolated with 10 µM NBQX in Mg^2+^-free medium) in representative recordings. *(Right)* Ensemble effects on AMPA-EPSC PPR and NMDA-EPSC amplitude (mean ± SEM). (**D**) Immunolabelling for α7-nAChR subunit in electron micrographs at the mossy fibre-granule cell synapse. An immunopositive pre-terminal mossy fibre (mf, bordered by arrows) opens up in a large bouton, which surrounds granule cell dendrites (*d*) (scale bar 0.5 µm). The inset shows a granule cell dendrite (*d*), contacted by a mossy fibre bouton (*b*), bearing an immunopositive post-synaptic specialisation (arrow). Scale bar 0.8 µm.

In whole-cell recordings from granule cells in acute cerebellar slices, local application of 1 µM nicotine increased the EPSCs evoked by mossy fibre stimulation by 19.4±5.4% (n = 56; p<0.001) (Fig. 1A). This effect was transient and the EPSC amplitude returned to baseline within 1–2 min, which is consistent with activation of a desensitising receptor subtype like the α7-nAChR [24]. Indeed, application of the selective α7-nAChR agonist, 10 mM choline, transiently increased the EPSCs (36.3±7.2%; n = 5; p<0.05) by an amount similar to that observed with nicotine. However, when co-applied with the α7-nAChR antagonist 10 nM MLA, 1 µM nicotine did not yield any increase, but rather caused a transient EPSC decrease (−24.1±12.0%, n = 4; p<0.01), and similar results were obtained when 10 nM MLA was applied alone (−27.6±3.8%, n = 5; p<0.05). Moreover, application of the α4β2-nAChR agonist 50 nM epibatidine did not cause any significant changes in synaptic transmission (−2.3±5.2%; n = 4; NS). This finding was confirmed by the fact that co-application of 1 µM nicotine with the α4β2-nAChR antagonist 1µM DHβE resulted in a potentiation similar to that caused by 1 µM nicotine alone (38.3±5.4%, n = 7; p<0.05).

Overall, these pharmacological experiments indicate that nicotine can regulate mossy fibre-granule cell synaptic transmission by activating α7-nAChRs that are sensitive to choline, insensitive to epibatidine, and blocked by MLA. The transient EPSC depression caused by MLA may indicate the presence of ambient acetylcholine in the slice. The specificity of the action of nicotine on α7-nAChRs was supported by recordings carried out in mice. In wild-type C56/BL6 mice, application of 1 µM nicotine increased the EPSCs evoked by mossy fibre stimulation by 50.1±13.2% (n = 4; p<0.05) (Fig. 1B). Conversely, application of 1 µM nicotine did not cause any significant EPSC changes in α7-nAChR KO mice (−1.1±7.5%; n = 4; NS) [34], [35] (Fig. 1B).

Nicotine at central synapses has been reported to act both pre- and postsynaptically [25], [36]. To determine the site of action of nicotine, we used a paired-pulse stimulation protocol and analysed the ratio between the 2^nd^ and 1^st^ peak in EPSC pairs (PPR) (Fig. 1C) [37], [38]. Moreover, we used different BAPTA buffer concentrations to control intracellular regulatory mechanisms, which can be activated by nAChR [11]. PPR was significantly reduced by nicotine application compared to control conditions (−36.8±5.7%; n = 8; p<0.005). The PPR change was not prevented by postsynaptic intracellular perfusion of 10 mM BAPTA (Fig. 1C; −39.3±11.5%, n = 6; p<0.05), supporting the involvement of a presynaptic mechanism. If enhancement of glutamate release is the only effect of nAChR activation, one should expect a similar increase in both AMPA and NMDA EPSC components, as already reported at this synaptic connection during forms of synaptic plasticity [39], [40]. However, in these recordings nicotine application was not found to significantly affect the NMDA current (−5.6±4.1%, n = 9; NS), except when 10 mM BAPTA was added to the intracellular solution (38.2±16.5%; n = 7, p<0.05). Thus, application of BAPTA unveiled a postsynaptic action of nicotine. This effect could be explained by the known desensitising action of Ca^2+^ on NMDA receptors [23], [41]. The BAPTA might have been acting by preventing Ca^2+^-dependent NMDA receptor desensitisation, thereby making the changes caused by nicotine in the AMPA and NMDA currents similar to each other (n = 7; p = 0.6; NS) (Fig. 1C).

The localisation of nicotinic receptor subunits was assessed by EM immunolocalisation. Immunolabelling for α7-nAChR at the glomerulus level was revealed both in pre- and in postsynaptic elements (Fig. 1D). Postsynaptically, immunoreactivity was associated with vesicular-like elements distributed both within the cytoplasm and adjacent to the plasma membrane, decorating postsynaptic specialisations in granule cell dendrites. Presynaptically, immunoreactivity was observed within the pre-terminal region of mossy fibres. Therefore, α7-nAChRs assembled in the plasma membrane have the potential to regulate mossy fibre-granule cell synaptic transmission, acting, as indeed indicated by electrophysiological data, through both pre- and postsynaptic mechanisms.

### α7-nAChRs are Required for Nicotine Facilitation of LTP Induction

**Figure 2:**
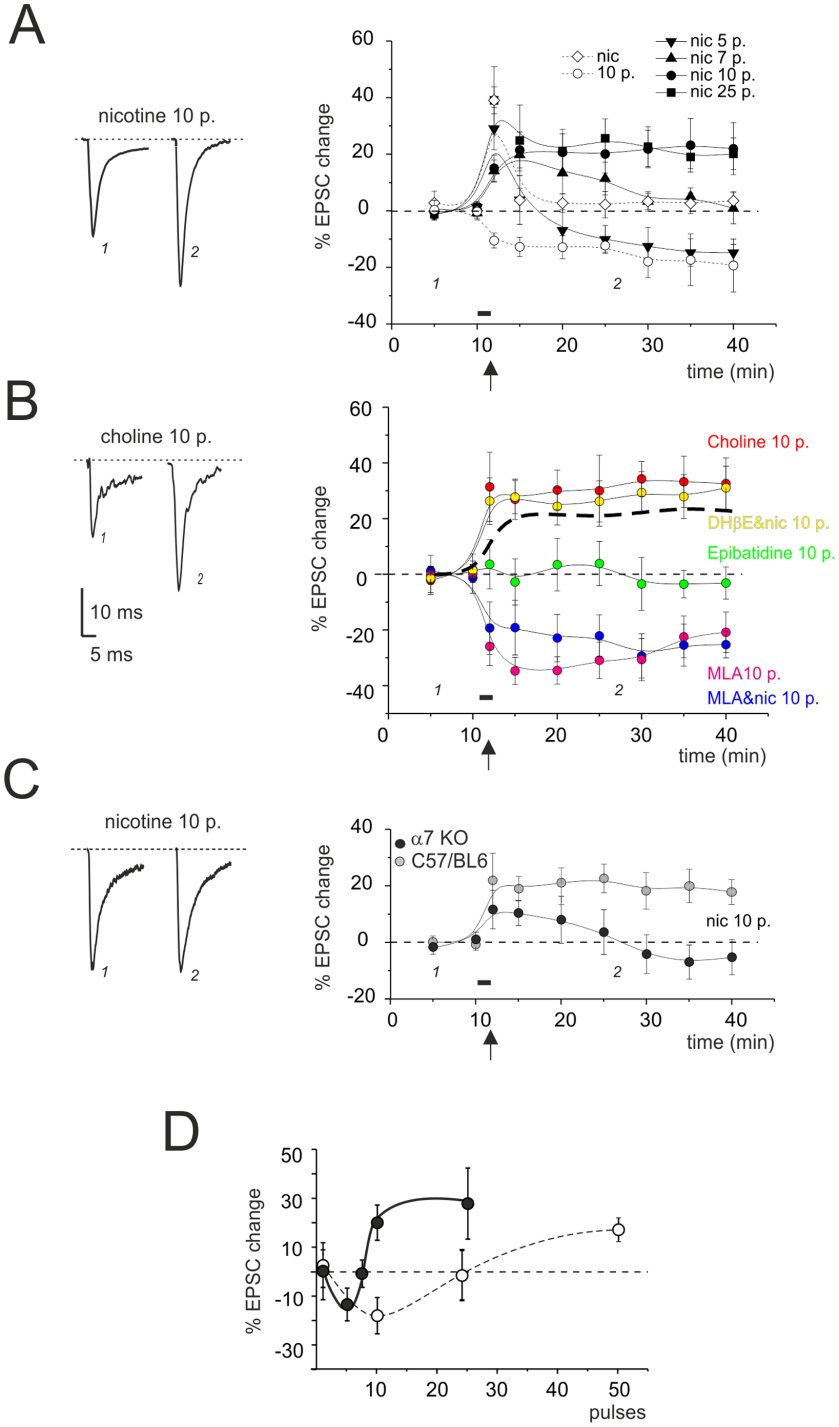
Facilitation of LTP induction by *α7-nAChRs*. In A, B, and C, patch-clamp recordings were performed from granule cells in cerebellar slices from the holding potential of –70 mV. Just after application of specific nicotinic agents for a duration of 100 sec, bursts of stimuli at 100 Hz were delivered to the mossy fibres while maintaining granule cells at –40 mV. The black bar indicates drug perfusion and the arrow indicates mossy fibre stimulation. (**A**) EPSC amplitude changes caused by bursts of different lengths following application of 1 µM nicotine. (*Left)* Average traces of 30 contiguous EPSCs taken from a representative experiment before and after induction (nicotine +10 pulses). *(Right)* Time course of EPSC amplitude changes (data points are mean ± SEM) when performing induction with a single pulse (n = 5), 5 pulses (n = 6), 7 pulses (n = 4), 10 pulses (n = 7), 25 pulses (n = 5). Mean ± SEM. (**B**) EPSC amplitude changes induced by a 10-pulse induction burst after application of different test solutions. These include Krebs’ solution either alone (n = 5) or dissolving different cholinergic agents: 10 mM choline (n = 4), 1 µM nicotine +10 nM MLA (n = 4), 50 nM epibatidine (n = 4), 10 nM MLA (n = 5), 1µM DHβE (n = 7). Same panel layout as in A. (**C**) EPSC amplitude changes in C57/BL6 (n = 4) and α7-nAChR KO mice (n = 4) after application of 1 µM nicotine with a 10-pulse induction burst. Same panel layout as in A. (**D**) EPSC amplitude changes at steady-state (20 min after induction) for different burst durations. The present recordings obtained following nicoptine application (closed symbols, continuous line) are compared with those obtained without nicotine application (open symbols, dashed line). Mean ± SEM (number of observations as indicated the text).

LTP and LTD have been observed at the mossy fibre-granule cell synapse following various patterns of activity [39], [40], [42]–[44]. The impact of nicotine on LTP induction was evaluated using 100 Hz tetani of different durations. In control recordings, 20 min after induction with a 100 Hz tetanus, LTD was observed with 10 pulses (−21.3±6.7%, n = 5; p<0.005) while LTP was observed with 50 pulses (20.4±4.5%, n = 5; p<0.005); the neutral point showed 25 pulses (−1.6±8.4%, n = 5; p<0.005) (cf. [42], [43]). The application of 1 µM nicotine (for 100 sec prior to the tetanus) also allowed the development of LTD and LTP conditional upon the number of pulses in the mossy fibre burst (Fig. 2A). Twenty minutes after induction, no net effect was observed with 1 pulse (0.2±11.6%, n = 5, NS), LTD was observed with 5 pulses (−13.5±6.7%, n = 6; p<0.01), a transient potentiation occurred with 7 pulses (−0.9±5.6%, n = 4, NS), and LTP was observed with 10 pulses (24.8±5.7%, n = 7; p<0.01) or 25 pulses (27.8±14.5%, n = 5; p<0.05). Therefore, with a 10-pulse train at 100 Hz, plasticity switched from LTD to LTP, although this depended on whether or not nicotine was perfused just before the tetanus, i.e. *nicotine facilitated LTP induction*.

The involvement of α7-nAChRs in LTP facilitation (cf. [26], [27], [45]) was investigated by applying specific receptor agonists and antagonists during the 100 sec prior to delivery of 10 pulses at 100 Hz (Fig. 2B). The application of 10 mM choline caused LTP (31.6±11.0%, n = 4, p<0.05) that was statistically indistinguishable from that caused by nicotine (p = 0.6; NS). Conversely, the co-application of 1µM nicotine with 10 nM MLA resulted in LTD (−24.1±9.3%, n = 4, p<0.05) indistinguishable from that observed with the same tetanus in the absence of drugs (p = 0.6; NS). Since the inhibition constant of MLA for α4β2-nAChRs (*K_i_* = 40 nM [46]) is 4 times higher than the actual concentration used, it can be assumed that MLA was acting almost exclusively though inhibition of α7-nAChRs). As a control condition, we also applied 10 nM MLA alone, and this again resulted in an LTD (−27.6±8.9%, n = 5; p<0.05) similar to that observed with MLA plus nicotine. Thus, α7-nAChR stimulation prior to a 100 Hz–100 ms tetanus is necessary and sufficient for LTP facilitation. Application of 50 nM epibatidine had no effect on plasticity (−5.7±8.2%, n = 4, NS). This is at odds with the LTD expected if epibatidine would have been completely ineffective. (LTD was indeed obtained with a 10-pulse train at 100 Hz without drug application as well as after application of nicotine and MLA). It seems unlikely that epibatidine acted on α7-nAChRs, since the inhibition constant for these receptors (*K_i_* = 233 nM [47]) is 5 times higher than the actual concentration used. To counter-test this effect, we co-applied 1 µM nicotine with the α4β2-nAChR antagonist 1 µM DHβE. In this case, we obtained an LTP similar to that caused by 1 µM nicotine alone (29.3±7.6%, n = 7; p<0.05). Therefore, heteromeric receptors, although not activated by single synaptic pulses (cf. Fig. 1A), might play a role in plasticity by preventing LTD generated with short 100 Hz tetani, but they do not interfere with induction of LTP.

The generality of nicotinic facilitation of LTP was assessed by running similar protocols in mice. In wild-type mice, which normally show LTP with long-train pulses [48], the application of 1 µM nicotine (for 100 sec prior to the tetanus) also allowed the development of LTP with 10 pulses (18.8±4.5, n = 4; p<0.05) (same induction protocol as for rat slices). The prominent role of α7-nAChRs was supported by the absence of any LTP facilitation in α7-nAChR KO mice [34], [35] following application of 1 µM nicotine (mean over 15 min: −10.6±5.9%, n = 4; Fig. 2C).

Overall, nicotine, primarily acting through α7-nAChRs, makes the LTP induction mechanism more efficient. The dependence of long-term synaptic plasticity on the number of pulses (Fig. 2D) was reconstructed from data reported in Fig. 2A and showed a Bienentock-Cooper-Munro (BCM)-like shape [49]. In the control, the neutral point was at around 25 pulses, whereas with nicotine it was at around 5 pulses. Thus, in order to achieve an LTP similar to that obtained in the presence of nicotine, trains in the control condition need to contain at least 50 pulses rather than just 10 pulses (Fig. 2D).

### Postsynaptic Induction and Presynaptic Expression of LTP during Nicotine Facilitation

**Figure 3:**
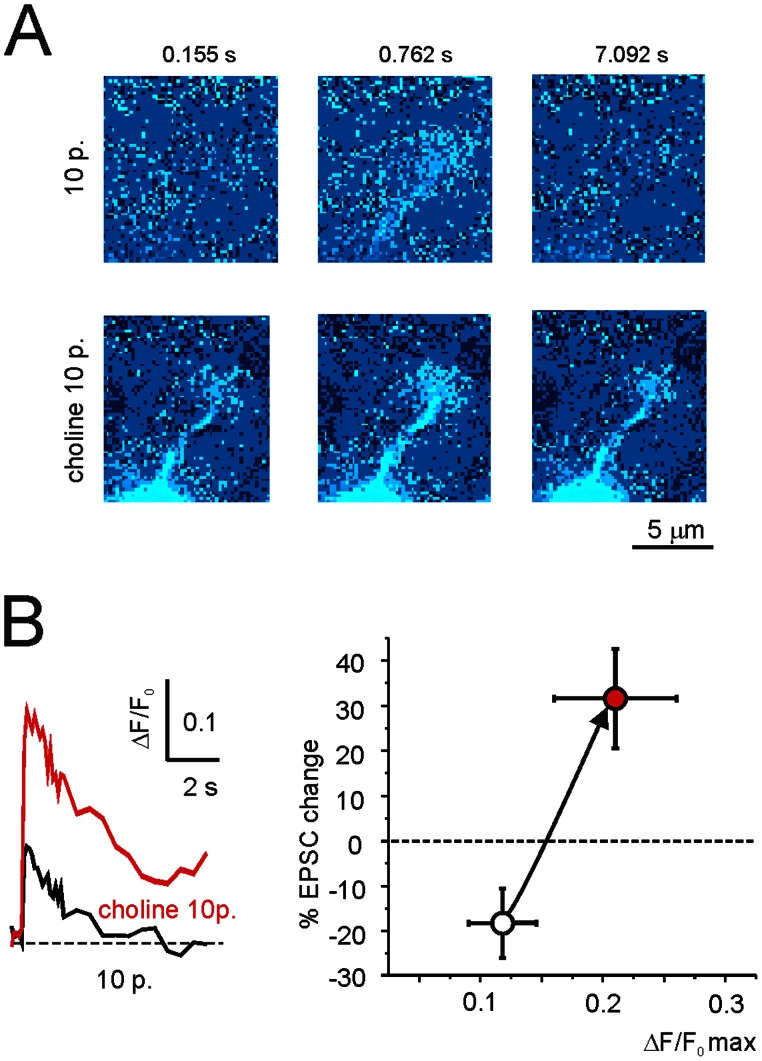
Postsynaptic induction of nicotine facilitated LTP through intracellular Ca^2+^ regulation. Intracellular Ca^2+^ concentration, [Ca^2+^]_i_, was measured in granule cell dendrites as OG1 relative fluorescence, ΔF/F_0_. Choline 10 mM was applied for 100 seconds just before a 10-pulse (100 Hz) mossy fibre burst, while holding the granule cell at –40 mV. **(A)** The sequence of pseudocolour images show higher Ca^2+^ increase in a granule cell dendritic ending when choline is perfused than in control recordings. **(B)** Time course of ΔF/F_0_ in recordings obtained in control and following choline perfusion (*left*) and relative EPSC changes (taken from Fig. 2) as a function of maximum ΔF/F_0_ during bursts of different duration (*right*). Choline moves the point (mean±SEM, n = 5) corresponding to the 10-pulse burst from LTD to LTP (arrow).

The induction of LTP at the mossy fibre-granule cell relay involves elevations in postsynaptic intracellular Ca^2+^ ([Ca^2+^]_i_) [42], [43]. In order to investigate the mechanism of LTP facilitation by nicotine, we performed calcium imaging experiments with Oregon Green BAPTA-1 (OG1) in the recording pipette (Fig. 3A). As reported previously, ΔF/F_0_ increased in the granule cell dendrites in response to a mossy fibre burst (10 pulses at 100 Hz). Following 10 mM choline perfusion (for 100 sec prior to the burst), the [Ca^2+^]_i_ peak increased significantly more than in the control condition (31.9±9.9%, n = 5; p<0.05; Fig. 3A,B). Moreover, the [Ca^2+^]_i_ transient became longer – the duration at half-peak amplitude, HW, increased by 46.6±7.6% (n = 5; p<0.05) –, increasing the [Ca^2+^]_i_ elevation (measured from the integral of the signals) by 67.4±6.8% (n = 5; p<0.05; Fig. 3B). Analysis of the relationship between synaptic plasticity and [Ca^2+^]_i_ (Fig. 3B) showed that when the induction occurred after choline perfusion, the point normally located in the LTD region moved into the LTP region. Thus, the α7-nAChR-dependent enhancement of [Ca^2+^]_i_ elevations in granule cells was correlated with the switch of plasticity from LTD to LTP, indicating that nicotine regulated the core postsynaptic induction process of granule cells. It should be noted that upon application of 10 mM choline or 1 µM nicotine, there was no observable change in the leakage current (at the time of maximum EPSC change: 6.2±5.8%, n = 10; NS), which suggests that the main effect of nicotinic receptors was not to modify cell electro-genesis, but rather to influence intracellular mechanisms, possibly through Ca^2+^ -dependent stimulation of Ca^2+^ release from intracellular stores (e.g. see [50]).

**Figure 4:**
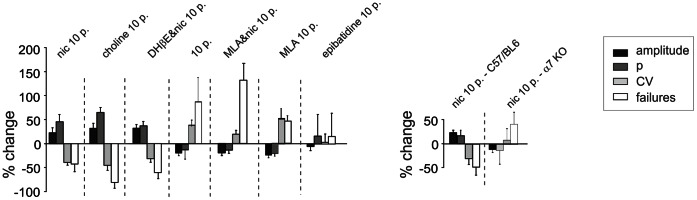
Evidence for presynaptic expression of nicotine facilitated LTP. Percent changes induced by 10-pulse bursts in EPSC amplitude, release probability (*p*), coefficient of variation (*CV*), and failure rate (*FR*) between 15 and 20 min after the induction of plasticity in the experiments A-C of Fig. 2 (p<0.01 for all parameters).

The expression of LTP at the mossy fibre-granule cell relay has been shown to reflect enhanced neurotransmitter release from mossy fibre terminals [40], [42]. The techniques and experimental conditions adopted in the present study were chosen to be identical to those used previously to investigate quantal transmission at this same synapse [37], [40]. In particular, in the present experiments the EPSCs were elicited by minimal mossy fibre stimulation (−31.7±2.0 pA; n = 27, failures excluded), which made it possible to evaluate statistical parameters (e.g. release probability) and the locus of plasticity changes by measuring peak amplitude fluctuations (coefficient of variation, CV) and failure rate (FR) (see, e.g., [37], [40], [43] and Methods; Fig. 4). Invariably, LTP facilitation by nicotine and choline was associated with a significant increase in release probability and a significant decrease in CV and FR, while the opposite occurred with LTD in controls and with MLA; furthermore, no significant changes occurred when neither LTP or LTD were observed with epibatidine, or when using α7-nAChR KO mice (data are summarised in Fig. 4). Overall, LTP and LTD expression patterns were similar to those observed in control conditions and were compatible with a presynaptic expression mechanism acting through a regulation of neurotransmitter release (cf. [42]).

### Increased Extension of LTP Over the Granular Layer during Nicotine Facilitation

**Figure 5:**
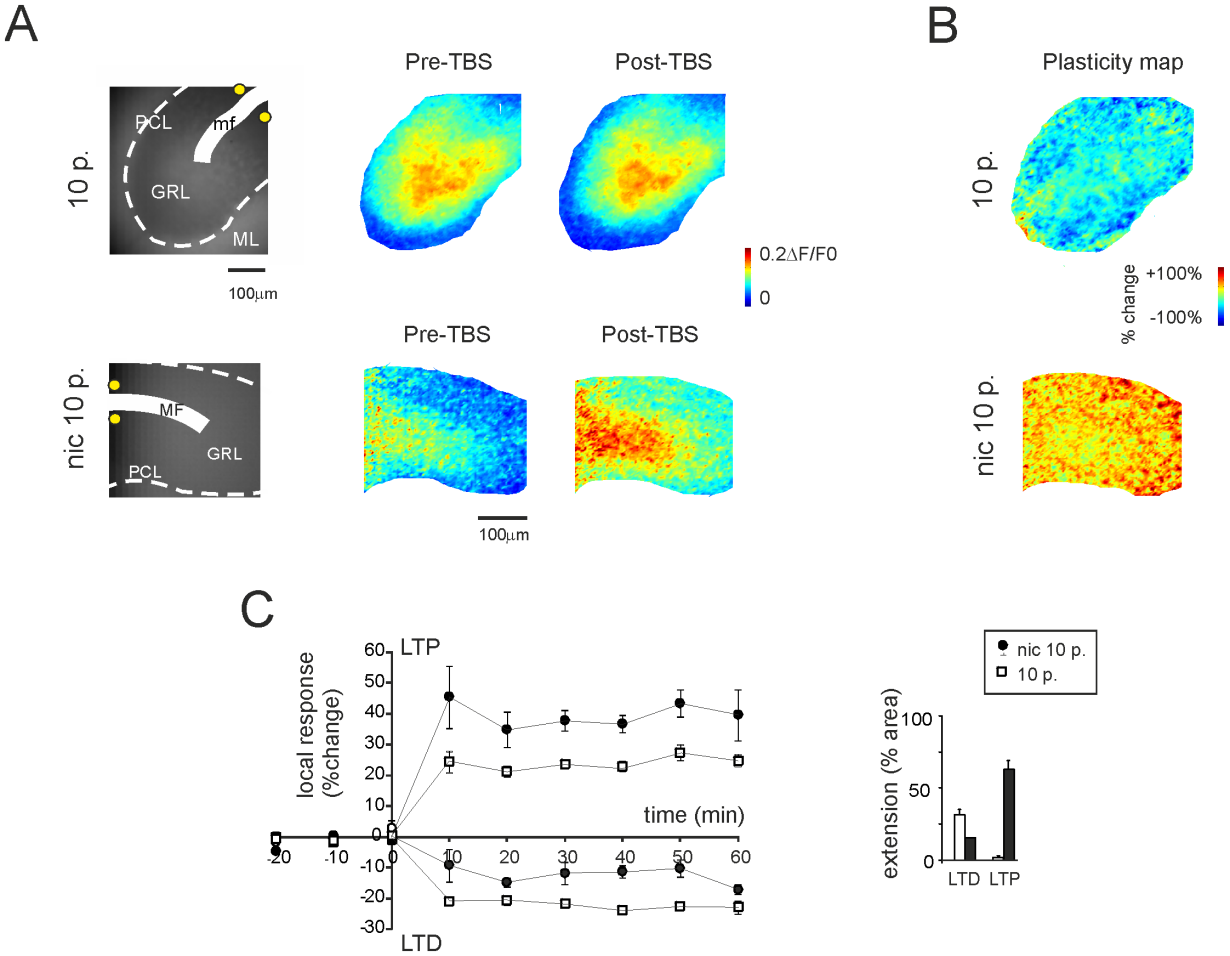
Saturation of LTP by nicotine application. VSD recordings were performed from the granular layer of cerebellar slices to measure the spatial organisation of the effect of nicotine following TBS. (**A**) Images of background epifluorescence in stained cerebellar slices show the granular layer (GRL), Purkinje cell layer (PCL), molecular layer (ML), mossy fibre bundle (mf), and position of the stimulating electrode (yellow dots). Coloured optical maps of granular layer activity evoked by a single mossy fibre pulse are compared before and after the induction of long-term synaptic plasticity by TBS. (**B**) The spatial distribution of LTP and LTD is shown in plasticity maps reporting the relative percent change in fluorescence after TBS. In control conditions, TBS induces more LTD than LTP, while, after application of 1µM nicotine, TBS induces extensive LTP, almost entirely the covering the granular layer. (**C**) Average time courses of all pixels showing either LTP or LTD, both in control condition and in the presence of 1µM nicotine. The histogram shows the relative area covered by LTP and LTD. A residual area showed no significant changes and does not appear in the bars, see Methods).

In response to theta-burst stimulation (TBS), LTP and LTD in the granular layer are usually distributed in a spatially organised manner, characterised by a prevalence of LTD over LTP [44]: in particular, when synaptic inhibition is blocked, the LTP/LTD ratio is increased. In the present experiments, LTP facilitation caused by nicotine was also expected to modify the LTP/LTD ratio in favour of LTP. We therefore applied TBS (8×10-pulse trains at 100 Hz every 250 ms) and detected synaptic plasticity changes using a voltage-sensitive dye (VSD) imaging system [51], [52] (Fig. 5A–B). In control conditions, VSD imaging revealed local response changes following TBS, which showed a similar intensity and time-course as LTP and LTD observed with multi-electrode array (MEA) local field potential recordings (LFPs) and whole-cell recordings (cf. [44]). As far as the spatial distribution of plasticity is concerned, in VSD recordings, LTP and LTD covered 15.3±4.5% and 31±5.5% of the surface (n = 5), respectively, whereas after 1 µM nicotine perfusion, they covered 76.4±7.8% and 0.09±0.02% of the surface, respectively (n = 5; p<0.01; Fig. 5C). Moreover, the average LTP intensity nearly doubled (from 24.1±1.1% in control conditions to 39.7±1.6% with nicotine; n = 5, p<0.01), while the average LTD intensity was reduced (from −22.1±0.7% in control conditions to −12.4±1.2% with nicotine; n = 5, p<0.01) (Fig. 5C). A greater proportion of neurons generating LTP in a given slice area could be advanced as a possible interpretation for this. Therefore, nicotinic facilitation drove the granular layer LTP toward saturation, as can be easily appreciated by comparing the plasticity maps in Fig. 5B.

### Nicotine Facilitation of LTP *in vivo*

**Figure 6:**
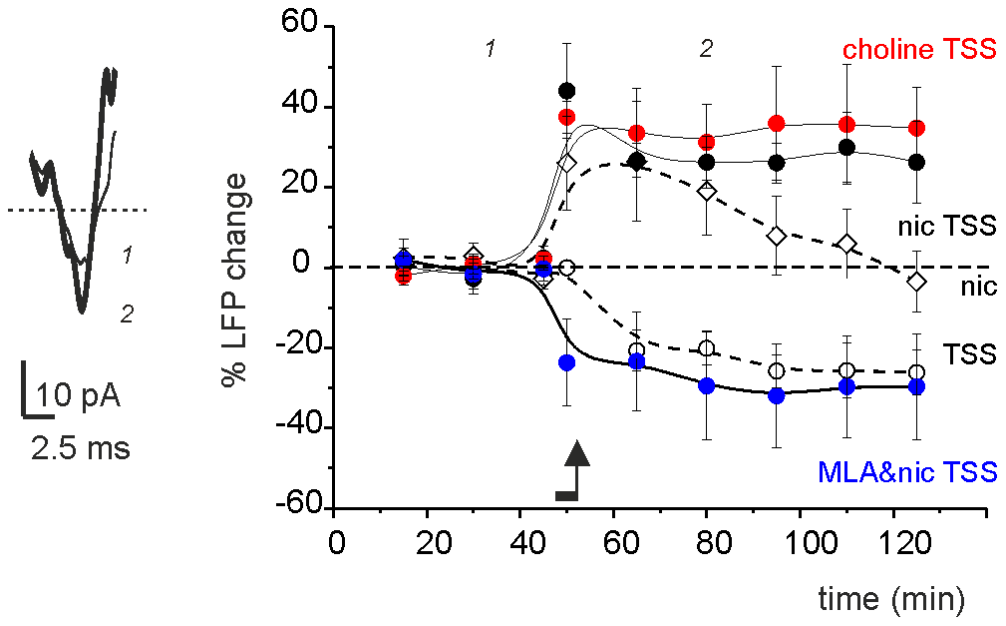
α7-nAChR stimulation facilitates LTP *in vivo*. LFP recordings were performed from the granular layer of the Crus-IIa of the right cerebellar hemisphere in response to air-puff stimulation of the homolateral whisker pad. A TSS air-puff induction pattern was delivered at the end of a 5 min microperfusion of drugs in different combinations: TSS+Krebs’ solution (n = 7), just 50 µM nicotine (n = 6), TSS +50 µM nicotine (n = 6), TSS +100 mM choline (n = 5), TSS +50 µM nicotine +0.5 µM MLA (n = 5). *Left*, LFPs recorded before and after TSS+nicotine (average of 100 traces). *Right*, time course of the LFP amplitude changes (mean±SEM). Drug microperfusion is indicated by a bar and TSS by an arrow.

Patterned sensory stimulation of the whisker pad has been shown to induce long-term synaptic plasticity in the granular layer of the anaesthetised rat [53]. To assess nicotinic effects *in vivo,* LFPs were performed in the granular layer (depth: 500 µm) of the Crus-IIa in anaesthetised rats using tactile stimulation of the whisker pad (Fig. 6) [53], [54]. A brief theta sensory stimulation (TSS; see Methods) was used to induce long-term synaptic plasticity [53]. In response to test pulses (0.1 Hz), LFPs remained at around baseline levels for at least 90 min (−1.9±2.5%, n = 9; data not shown). Conversely, a brief nicotine intracerebellar microperfusion caused a transient LFP increase, which reached a maximum amplitude of 26.0±11.6% (n = 5; p<0.05) in 15 min, before declining to 5.9±8.6% (n = 5) of baseline level over the following 60 minutes. TSS *per se* induced a persistent LFP reduction (−25.6±6.8% after 60 min, n = 7; p<0.05), indicating induction of LTD. However, TSS delivered at the end of nicotine (50 µM for 5 min) or choline (100 mM for 5 min) micro-injection caused LTP. The LTP facilitation by nicotine (30.0±8.8% after 60 min, n = 6; p<0.01) vs choline (37.4±14.9% after 60 min, n = 5; p<0.005) was similar (p = 0.8). Conversely, TSS delivered just after the end of MLA and nicotine co-injection (0.5 µM and 50 µM for 5 min, respectively) induced LTD (–25.7±15.6% after 60 min, n = 5; p<0.05). The LTD obtained with nicotine and MLA was indistinguishable from that obtained in control conditions (p = 0.9; NS). In all cases, LTP and LTD corresponded in amplitude and time course to those previously reported in this same preparation [53]. Therefore, the documented effects on LFP amplitude depended on the activation of α7-nAChRs and followed the same pharmacological pattern observed in slice recordings, which suggests that the nAChR-mediated regulatory processes observed *in vitro* also occurred *in vivo*.

### Nicotinic Receptor Stimulation Affects Vestibulo-ocular Reflex Adaptation

**Figure 7:**
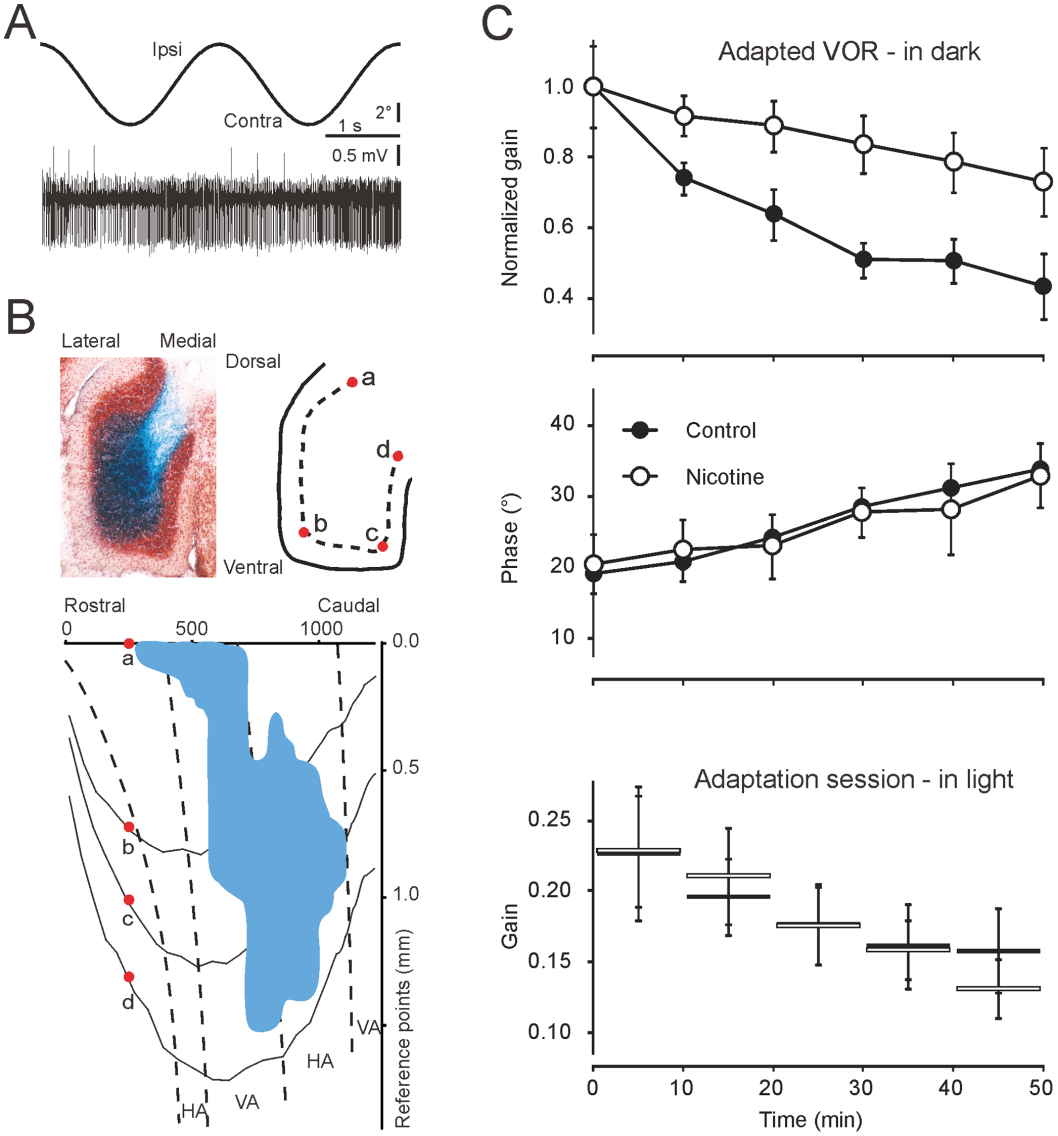
Nicotine microperfusion impairs VOR gain adaptation. The flocculus of the mouse was located using extracellular electrophysiological recordings during visual stimulation. (**A**) Increased complex spike activity during horizontal, contralateral movement of the visual field confirmed the location of vertical axis (VA) Purkinje cells. Based on the polarity of the waveform of Purkinje cell activity identified in each track, the location of the granule cell layer was determined, and a dye-labelled vehicle solution with or without 5–10 ng nicotine (n = 8 and n = 7, respectively) was injected. (**B**) Dye diffusion was analysed histologically and compared with the unfolded (according to points a-d) mouse floccular map [72]. Only injections covering at least 10% of the VA were included. The effect on motor learning was assessed by subjecting mice, 15 min after injection, to five 10 min sessions of in-phase vestibular and visual input, aimed at decreasing the VOR gain. (**C**) Mice injected with nicotine showed a significantly impaired ability to decrease their VOR gain. Nicotine did not affect the timing (phase) of the VOR, and the gain during the training was also not affected, indicating that the deficit is specific for adaptation rather than performance.

Given the remarkable impact of nicotinic receptor activation on granular layer long-term synaptic plasticity, we wondered whether nicotine application might modify cerebellar motor learning. In order to adapt to novel input patterns, plasticity probably needs to take place at specific synapses in a spatially organised manner [4]. On the basis of this reasoning, we predicted that pharmacological application of nicotine, by saturating plasticity toward LTP, would alter the normal adaptation behaviour of the vestibulo-ocular reflex (VOR). To test this hypothesis, the part of the flocculus responding to stimulation around the vertical axis area (VA area) was localised, nicotine was microperfused locally into the granular layer, and VOR adaptation [55], [56] was monitored (Fig. 7A). Mice were subjected to in-phase visual and vestibular stimulation, aimed at decreasing their VOR gain. Diffusion of nicotine was visualised by post-mortem 3D-to-2D reconstruction of the distribution of a simultaneously co-perfused dye (Fig. 7B, see Methods). Data were included in the test group only when perfusion still revealed an injection volume greater than 10% of the granular layer underlying the VA area about 1.5 hr after injection. Preparations with substantial diffusion of the dye into the molecular layer were excluded (see Methods). In saline-injected controls, VOR adaptation was similar to that reported previously [55], indicating that electrode insertion and injection of solution into the flocculus did not, by themselves, alter the VOR. Compared with controls, VOR gain adaptation was impaired following nicotine application (Fig. 7C). After 50 min of adaptation, VOR gain decreased by 56±9% (n = 8) in time-matched saline-injected controls and by 27±10% (n = 7) after nicotine perfusion (p = 0.01, repeated measures ANOVA). The injections of nicotine did not affect the VOR phase during adaptation (p = 0.91, repeated measures ANOVA). Nicotine also had no impact on the absolute VOR gain before training (data not shown), or on the gain during the 10 min training sessions in the light (p = 0.98, repeated measures ANOVA), indicating that the effects on adaptation are not caused by deficits in motor performance. Therefore, we conclude that nicotine micro-injection into the granular layer significantly impairs VOR adaptation.

## Discussion

The main observation emerging in this paper is that cholinergic agents enhance LTP at the cerebellum input stage through activation of α7-nAChRs. Nicotinic regulation enhanced LTP elicited by mossy fibre burst in cerebellar slices as well as LTP elicited by sensory stimulation *in vivo*. These effects were accompanied by a modification of the overall LTP/LTD balance in the granular layer network and were able to modify VOR adaptation. These results suggest that nicotinic facilitation of LTP at the input stage of the cerebellum provides an effective mechanism for controlling cerebellar learning and functioning.

### Nicotinic Receptor Activation in the Cerebellum Granular Layer

The prominent involvement of the α7 subtype of nAChR on neurotransmission was indicated by the similar potentiating effect of choline and nicotine, by the inhibitory effect of MLA, by the inefficacy of epibatidine and DHβE [11], and by the absence of nicotine effects in α7-nAChR KO mice [34], [35]. It is worth noting that sustained application of 1µM nicotine is expected to cause rapid (∼1 sec) desensitisation of α7-nAChRs, which display an IC50 for nicotine at about 0.85 µM [11]. However, the fact that MLA prevented the effect of nicotine and that desensitisation took minutes to become established indicated that nicotine concentration at the synapse was below 1 µM, implying that nicotine was acting through receptor activation rather than desensitisation. Indeed, desensitisation was found to have little influence on the induction of LTP either *in vitro* or *in vivo*. One possibility is that activation of α7-nAChRs modified intracellular mechanisms (like Ca^2+^ release from the stores) just before the induction train, as suggested by STDP experiments in the hippocampus [30].

Immuno-electron microscopy revealed α7-nAChR subunits both in mossy fibre terminals and in granule cell dendrites. Presynaptically, α7-nAChRs enhanced neurotransmitter release probability, as indicated by AMPA current PPR decrease [38]. Consistent with this, α7-nAChRs promote the release of [(3)H]-glutamate [22]. Postsynaptically, α7-nAChRs enhanced dendritic [Ca^2+^]_i_ elevations during repetitive synaptic transmission [42], [43]. As regards LTP induction, α7-nAChR pharmacology corresponded to that observed during low-frequency mossy fibre-granule cell transmission, except for the fact that epibatidine prevented the LTD normally observed with short-stimulus trains in control recordings. Thus, we cannot rule out the possibility that a role in regulating the LTP/LTD balance, albeit more subtle, is also played by heteromeric nicotinic receptors.

### Nicotinic Facilitation of Mossy Fibre-granule Cell LTP

It was previously shown that LTP induction at the mossy fibre-granule cell synapse is underpinned by NMDA receptors and potently enhanced by Ca^2+^-induced Ca^2+^ release (CICR) from intracellular stores [43]. It is highly improbable that α7-nAChRs contributed to the AMPA receptor-mediated depolarisation (glutamate receptor blockers fully abolish mossy fibre-granule cell EPSC [33], [57]), or that they enhanced the NMDA receptor-mediated Ca^2+^ influx (the NMDA current was reduced through a Ca^2+^- dependent mechanism, see [41], [58]). Instead, α7-nAChRs could contribute directly to Ca^2+^ elevations in sub-membranous microdomains and, by increasing glutamate release, may favour mGluR activation and potentiate CICR [42], [43]. It is thus possible that enhanced [Ca^2+^]_i_ increase in granule cell dendrites, combined with enhanced glutamate release, generates a coordinated set of processes facilitating LTP induction.

The functional relevance of the nicotinic gating effect is supported by the observation that nicotinic agents were able to discriminate LTP vs LTD *induction* over the restricted number of spikes (between 5 and 10) typically conveyed by mossy fibre bursts [59]. Furthermore, nicotine shifts the plasticity set-point so that short isolated bursts, which would normally induce LTD, readily induce LTP. Nicotinic gating conforms to the “sliding threshold” mechanism predicted by the BCM plasticity rule [49], in that the shape of the Ca^2+^-plasticity plot is not changed but its neutral point is shifted. Unlike nicotine-facilitated induction, nicotine-facilitated LTP *expression* occurs through increased neurotransmitter release (shown by a decrease in AMPA current PPR, failures and CV), and thus exploits the expression mechanisms already reported at this synapse [40]. Taken together, these mechanisms of action suggest that nicotine facilitates LTP through a true regulation of existing mechanisms. Interestingly, α7-nAChR effects did not involve NMDA current changes, suggesting that the LTP set-point could be regulated without altering the NMDA receptor-dependent control over spike burst transmission [5]–[7].

### The Impact of Nicotinic Facilitation of LTP *in vivo*

The LTP facilitation processes observed *in vivo* and *in vitro* shared the same pharmacological selectivity for α7-nAChRs and were therefore probably similar. The final effect of LTP on circuit functions is thought to depend on the arrangement of synaptic weights in the network [4], [60], [61]. Nicotinic facilitation extended the granular layer surface over which LTP could be observed, which suggests that it could allow synaptic inputs to drive the local synaptic population toward saturation. Pharmacological application of nicotine, by saturating granular layer LTP, was expected to disrupt the LTP/LTD balance [43], [44], [56], disabling the related adaptation mechanisms. Indeed, nicotine microperfusion impaired VOR gain adaptation, which has recently been reported to require intact granular layer synaptic plasticity [56]. The absence of α7-nAChR-dependent LTP facilitation could also explain the impairment of trace eyeblink conditioning and procedural learning revealed in α7-nAChR KO mice [34], [35]. In natural conditions, α7-nAChR activation may be underpinned by activity of pedunculo-pontine tegmental nucleus (PPTN) neurons, which are major providers of ACh to the cerebellum [62], [63], and could relay appropriate attentional and cognitive signals for gating cerebellar learning. Further experimental studies are needed to clarify these behavioural and pharmacological implications.

### Conclusions and Functional Implications

Taken together, the present results indicate that the cholinergic system gates LTP at the cerebellar mossy fibre-granule cell synapse [3]. This observation opens up the way for a reinterpretation of one of Marr’s major predictions [8] and its subsequent integration into the adaptive filter model [9]. Marr explicitly excluded the possibility that mossy fibre-granule cell synaptic weights could be modified by activity, noting that “*sooner or later all weights would be saturated*” (and plasticity would therefore have served no useful purpose) [6]. Our results suggest that the LTP/LTD balance at this synapse, provided the distribution between LTP and LTD remains finely tuned, could play an important role in controlling adaptive behaviours. The cholinergic mechanism could be activated through α7-nAChRs, which are critical for enhancing cognitive processes and for facilitating attention and memory. On this basis it can be suggested that this mechanism links cerebellar learning to such behavioural states on a global brain processing scale. We speculate that nicotinic agents specifically targeted toward α7-nAChRs may be beneficial for the treatment of cerebellar ataxia [27] and autism (which has been shown to have a cerebellar component [14], [17]).

## Materials and Methods

All experiments were conducted in accordance with international guidelines on the ethical use of animals (European Community Council Directive 86/609/EEC).

### Cerebellar Slices

Acute 220 µm thick slices were obtained from Wistar rat [42] cerebella on postnatal day P18-P23. When specified, slices were also prepared from C57BL/6 mice and strain-matched α7-KO mice [34], [35]. Slice cutting was performed in ice-cold K-gluconate-based solution followed by incubation at 32°C in oxygenated Krebs’ solution for at least 1 hour before recording. In order to isolate NMDA currents, in some experiments slices were incubated in Mg^2+^-free Krebs’ solution, which was then perfused along with 2 µM NBQX [2,3-Dioxo-6-nitro-1,2,3,4-tetrahydrobenzoquinoxaline-7-sulfonamide (Tocris Bioscence, Bristol, UK)] in the recording chamber.

### Nicotine Receptor Pharmacology

(−)-Nicotine hydrogen tartrate salt minimum 98% TLC (1 µM), choline chloride [(2-Hydroxyethyl)trimethylammonium chloride] (10 mM), and MLA [Methyllycaconitine citrate hydrate] (5 nM) were obtained from Sigma-Aldrich, and epibatidine [(±)-exo-2-(6-Chloro-3-pyridinyl)-7-azabicyclo[2.2.1.]heptane] (50 nM) and DHβE [(dihydro-β-erythroidine hydrobromide)] (1 µM) from Tocris Bioscience. Stock solutions were prepared for all drugs and stored frozen at –20°C. Drugs were diluted to their final concentration in Krebs’ solution before use and locally applied through a two-barrel pipette positioned 50–100 µm from the recorded cell. In all experiments, after a 10 min control period, short-lasting perfusion of drugs (1.5 min) was started [11].

### Electrophysiology

Slices were transferred to the recording chamber on the stage of an upright microscope (Zeiss F2S, Oberkochen, Germany) and perfused at 1.5 ml min^−1^ with oxygenated Krebs’ solution containing 10 µM bicuculline methobromide (ABCAM, Cambridge UK) and maintained at 32°C by means of a Peltier feedback device (TC-324B, Warner Instruments Corp., Hamden, CT, USA). Recordings were obtained in the whole-cell configuration using a Multiclamp 700B amplifier (−3dB; cut-off frequency = 10 kHz). Signals were digitised at 20 kHz using pClamp 9 and a Digidata 1322A A/D converter (Molecular Devices, Union City, CA, USA). Patch pipettes were pulled from borosilicate glass capillaries (Hilgenberg, Malsfeld, Germany) and filled with the following solution (values in mM): 81 Cs_2_SO_4_, 4 NaCl, 15 HEPES, 15 glucose, 2 MgSO_4_ · 7H_2_O, 0.1 BAPTA-free, 0.05 BAPTA-Ca^2+^, 3 Mg^2+^-ATP, 0.1 Na^+^-GTP, pH 7.2 adjusted with CsOH. This solution maintained resting free [Ca^2+^] at 100 nM and had a resistance of 5–7 MΩ before seal formation. The stability of patch-clamp recordings was monitored by analysing current relaxation induced by a 10 mV step from a holding potential of –70 mV [42]. Transient current analysis yielded a membrane capacitance (C_m_) of 3.7±0.1 pF (n = 72), membrane resistance (R_m_) of 2.5±0.1 GΩ (n = 72), and series resistance (R_s_) of 19.0±0.8 MΩ (n = 72). The 3-dB cell plus electrode cut-off frequency was *f*_VC_ = (2πR_s_C_m_)^−1^ = 2.6±0.09 KHz (n = 72) and did not significantly change during recordings. Mossy fibres were stimulated with a bipolar tungsten electrode (Clark Instruments, Pangbourne, UK) via a stimulus isolation unit at 0.1 Hz (paired-pulse stimulation was delivered at 20 ms inter-pulse intervals in some cases) [40]. Long-term synaptic plasticity was induced by continuous stimulation (one burst with a duration ranging from 50 to 250 ms at 100 Hz) while stepping the membrane potential from –70 mV to –30 mV.

### EPSC Analysis

EPSC amplitude was measured as the difference between EPSC peak and the current level just before stimulation. The PPR between the first and second EPSC in a sequence was PPR = IPSC_2_/IPSC_1_. For quantal analysis, minimal EPSCs were obtained by finely tuning the stimulation intensity [37], [40] and quantal parameters of release were obtained by using three statistical methods deriving from binomial statistics [64], [65] [40], [66].

### Quantal Analysis of Neurotransmission

The techniques and experimental conditions used in this study were designed to reproduce those already used to investigate quantal transmission at this same synapse [37], [40]. In previous papers, cross-validation of data obtained from the analysis of minis, multiple synapse recruitment, AMPA and NMDA receptor currents, spillover currents, paired-pulse facilitation, CV and failure analysis at different calcium concentrations made it possible to exclude spurious effects due to silent synapses, which are not present at the mature mossy fibre-granule cell relay [37], [40]. The results were confirmed by Ca^2+^ imaging [37], [40] and modelling [37], [40]. As shown in those papers, variations in CV, FR and PPR ratio give a reliable assessment of the synaptic changes occurring during the expression of long-term synaptic plasticity, allowing estimation of changes in release probability. These methodological aspects are briefly summarised here (for more detail, readers are referred to [40]).

For quantal analysis, minimal EPSCs were obtained by finely tuning the stimulation intensity until responses jumped from zero to a definite level [37], [40]. Responses lacking both direct and indirect response components were identified as stimulation failures and eliminated. The error introduced in EPSC CV by the indirect response, which causes non-zero failures, was eliminated by setting all indirect responses to zero (see [40]). The overlap between the CV and failure methods in predicting release probability provided support for the effectiveness of this method. Moreover, the effectiveness of minimal EPSC analysis was further supported by the fact that the EPSC size and the estimated number of release sites coincided with that previously reported for unitary synapses in the rat. For this analysis, we assumed that quantum properties do not change in plasticity experiments and used quantal parameters previously determined for this same preparation and these same recording conditions [40].

In order to improve the reliability of the parameter estimation, the quantal parameters of release were obtained by using three statistical methods corresponding to eq.1, eq.2 and eq.3. These methods derive from binomial statistics and are only schematically described here, since they do not significantly differ from previous applications (for comprehensive information, see [64], [65] [40], [66]).

The quantal theory states that the mean number of quanta released at each impulse (*m*, mean quantum content) depends on the number of releasing sites (*n*) and on the probability (*p*) that each quantum (*q*, quantum size) will be released, so that EPSC variance (*S^2^* = SD^2^) and mean amplitude (*M = mq)* are related through a parabolic function and EPSC variability depends on the number of released quanta. The contribution of intrinsic quantum variability can be accounted for by identifying intra-site (type-I) and inter-site (type-II) sources. Intra-site variability (*cv*_I_) reflects fluctuations in the number of open channels at single sites (*cv*_I-ss_) and asynchrony in quantal delay at the EPSC peak (*cv*_I-qd_). Inter-site variability (*cv*_II_) reflects differences between postsynaptic densities. Thus, the total quantal variance at the EPSC peak can be expressed as *cv^2^*_tot_ = *cv^2^*_I_+*cv^2^*_II_ = (*cv^2^*_I-ss_+*cv^2^*_I-qd_)+*cv^2^*_II_. The variability of mEPSCs (*cv_q_*) includes intra-site and inter-site quantal variability, *cv^2^_q_* = *cv^2^*_I-ss_+*cv^2^*_II_. Since *cv^2^*_I-ss_ is typically similar to *cv^2^*_II_, we divided the two terms equally as *cv^2^_I-ss_*≈ *cv^2^_II_* = ½ cv^2^_q_ (this assumption is discussed in [67]). The term *cv*_I-qd_ was obtained by measuring the difference in variance associated with stimulus-aligned EPSCs compared with onset-aligned quantal EPSCs in low-Ca^2+^ solutions (as proposed by [67]). Thus, *cv^2^*_I_≈½ cv^2^_q_ *+cv^2^*_I-qd_ and *cv^2^*_tot_ ≈cv^2^_q_ *+cv^2^*_I-qd_.

The relationship between EPSC *S^2^* and *M*, constructed by using different Ca^2+^ concentrations in the extracellular solution, was analysed using a binomial model under the assumption of homogeneous release probability (this was supported by the substantial symmetry of the data distribution, see [67])
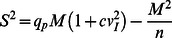
(1)


The model can also be applied without knowledge of the whole variance/mean distribution [64], since the parameters *p* and *n* can be calculated from the mean amplitude and coefficient of variation of EPSCs (*M = mq_p_* and *CV = S/M,* where *S* is EPSC SD). In this model *m* = *np*, SD^2^ = *np*(1−*p*), and the probability *p* is:
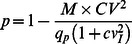
(2)


An estimate of *p* could also be obtained from the FR (N_0_/N; N_0_ are the failures out of N responses), which does not explicitly depend on previous determinations of quantum properties except that the number of releasing sites needs to be calculated beforehand with eq.1 or eq. 2:
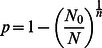
(3)


Although the three methods have different dependencies on experimental measurements and different intrinsic estimation errors [64], they yielded very similar parameter values, thereby supporting the reliability of *p* estimates.

The release probability, *p*, calculated from CV was 0.50±0.02 (n = 7), and that calculated from FR was 0.56±0.06. These values, which were similar to those reported previously in this same preparation [40], [42], increased during LTP by 47.8±5.6% (n = 7, p<0.005) and by 31.4±1.6% (n = 7, p<0.001), respectively. Therefore, not only were the two *p* estimates similar, they also showed a similar variation, supporting the consistency of quantal analysis.

### Calcium Imaging

Calcium imaging was performed, as previously reported in this same preparation, [43] by using Oregon green BAPTA-1 (OG1; Invitrogen Life Science, CA, USA). For experiments combining voltage-clamp and fluorescence Ca^2+^ imaging, whole-cell recording pipettes were filled with the following solution (values in mM): 140 CsCl, 4.6 MgCl_2_, 10 HEPES, 4 Na^+^-ATP, 0.4 Na^+^-GTP and 0.2 OG1 in place of the BAPTA/Ca^2+^buffer. Granule cells were identified with a ×63, 0.9 NA water immersion objective (Olympus, Hamburg, Germany). Digital fluorescence images were obtained using an excitation light source from T.I.L.L. Photonics (Planegg, Germany) controlled through Axon Imaging Workbench AIW5.2 (INDEC Systems). During recordings, images were acquired with a 50 ms exposure/image at video rate. The background-subtracted fluorescence F(n) = f(n)−B(n) was then used to evaluate ΔF/F_0_(n) = (F(n)−F_0_)/F_0_, where F_0_ is the average background-subtracted resting fluorescence over four consecutive images before stimulus application. Analysis of images was performed with AIW-5.2 software.

### Extracellular Recordings *in vivo*

Extracellular field recordings were performed in the granular layer of the Crus-IIa in 20–24-day-old Wistar rats (internal breeding, Harlan). The animals were deeply anesthetised with intraperitoneal urethane injection (1.4 mg/kg; Sigma-Aldrich) and vital signs were constantly monitored [53]. Animals were placed on a stereotaxic table (David Kopf Instruments, Tujunga, CA, USA) and body temperature was maintained at 38±0.5 C through a feedback temperature controller (Fine Science Tools Inc; Foster City, CA, USA). A craniotomy was made over the right hemisphere (AP −13 mm ML 3 mm). The dura mater was carefully removed and extracellular Krebs’ solution was placed on the cerebellar surface [54], [68], [69] [53]. LFPs from the granular layer were obtained at a depth of 500 µm with glass borosilicate pipettes (Hilgenberg) filled with NaCl 2M. Sensory stimulation was performed using a plastic pipette connected to an MPPI-2 Pressure Injector (Applied Scientific Instrumentation, Eugene, OR, USA) and positioned 2–3 mm from the whisker pad. Air puffs were delivered at 0.1 Hz (30 ms, 40 psi). TSS was used to induce long-term synaptic plasticity (4-Hz stimuli delivered for a duration of 2.5 sec). Extracellular currents were recorded with Multiclamp 700A amplifier (Molecular Devices), band-pass filtered between 100 Hz (high-pass) and 2 KHz (low-pass), digitised at 50 KHz using a Digidata 1322A interface, and stored on a PC using Clampex 8 (Molecular Devices). Drugs were dissolved in Krebs’ solution, placed in a microlitre syringe (Hamilton, Bonadus, Switzerland) and injected 500 µm deep into the Crus-IIa of the cerebellar cortex close to recording electrode through a controlled micro-pump. Nicotine (50 µM) was perfused at a rate of 0.2 µl/min for 5 minutes while choline chloride (100 mM) was perfused at 1 µL/min for 5 minutes. MLA (0.5 µM) and nicotine were co-perfused at 0.2µl/min for 5 minutes. Extracellular signals were processed off-line with Clampfit 9.2 (Molecular Devices). The signal-to-noise ratio was improved by averaging 100 consecutive traces and by digital filtering (Gaussian low-pass filter 1 kHz). Waveform peak amplitudes were measured relative to the preceding 10 ms baseline and delays were measured between the onset of the stimulus and the peak of the waveform.

### VSD Imaging

The VSD imaging procedures have been reported in previous papers [44], [51], [52]. The slices were incubated for 30 min in oxygenated Krebs’ solution supplemented with 3% Di-4-ANEPPS mixed with 50% foetal bovine serum (Molecular Probes). The dye (Di-4-ANEPPS, Molecular Probes) was dissolved and stocked in Krebs’ with 50% ethanol (Sigma-Aldrich) and 5% Cremophor EL (Sigma-Aldrich). After thorough washing, the slices were transferred to the recording chamber and superfused with standard extracellular solution (2–3 ml/min) maintained at 32°C by means of a feedback temperature controller (Thermostat HC2, Multi Channel Systems, Reutlingen, Germany). The mossy fibres were stimulated through a bipolar tungsten electrode using an isolated stimulator (STG 1008, Multi channel systems). Slices were observed with an upright epifluorescence microscope (BX51WI, Olympus, Europa GmbH, Hamburg, Germany), equipped with a 20X (XLUM Plan FL 0.95 NA). The light generated by a halogen lamp (150W, MHF-G150LR, MORITEX Corp., Tokyo, Japan) was controlled by an electronic shutter (model0, Copal, Co., Tokyo, Japan) and then passed through an excitation filter (λ = 530±10 nm), projected onto a dichroic mirror (λ = 565 nm). Fluorescence generated by the tissue was transmitted through an absorption filter (λ>590 nm) to the CCD camera (MICAM Ultima, Scimedia, Brainvision, Tokyo, Japan). The whole imaging system was connected through an I/O interface (Brainvision) to a PC controlling illumination, stimulation and data acquisition. Data were acquired and displayed by Brainvision software and signals were analysed using routines written in MATLAB (Mathworks, Natick, USA). In order to generate plasticity maps, signals were simultaneously detected and analysed in all the pixels of the granular layer. LTP and LTD were identified as positive or negative variations with respect to the average values of the pre-induction period, and the percentages of areas showing LTP or LTD were calculated with respect to all stable pixels of the granular layer. Pixels were considered stable only when their difference from the average was lower than two times the standard deviation. Moreover, only variations greater than ±10% and persistent throughout the recording were considered in the calculation of areas showing LTP or LTD.

### Immuno-electron Microscopy

P16-P30 rats were perfused through the ascending aorta with an oxygenated Ringer’s solution, followed by a fixative composed of 4% freshly depolymerised paraformaldehyde and 0.1% glutaraldehyde in 0.1 M phosphate buffer (PB), pH 7.4. Brains were removed, post-fixed overnight at 4°C and the cerebellum was cut using a vibratome into 60 µm thick sagittal sections, collected free floating in 0.1 M PB. After blocking the endogenous peroxidases and non-specific binding sites, slices were incubated in a rabbit anti-α7nAChR subunit antibody (Abcam, Cambridge, UK, ab23832) diluted 1∶300 in 1% DM in 0.1 M PB. Antigen-antibody binding sites were revealed by the peroxidase-anti-peroxidase method: after rinsing in PB, slices were incubated first in 1∶100 goat anti-rabbit IgG (1 h at RT) (Covance, Emaryville, CA, USA); they were then rinsed in PB again and incubated in 1∶300 Activity Select rabbit PAP (Covance) (1 h at RT). [70] [71]. Diaminobenzidine (DAB)- H_2_O_2_ was used as the chromogen to reveal antibody binding sites. After the DAB reaction, sections were rinsed, osmicated and dehydrated (at 4°C). During dehydration, *en bloc* staining for 20 min with 1% uranyl acetate in 70% ethyl alcohol was performed. Sections were flat embedded in Epon 812 epoxy resin. Ultrathin sections (60 nm thick) were cut using a Reichert ultramicrotome, collected on copper grids, lightly counterstained with 0.2% lead citrate to avoid shading of the immunoreaction product, and viewed under a Philips EM208S transmission electron microscope at 60 kV. Digital images were acquired with a Megaview III camera and adjusted for light and contrast by using the Adobe Photoshop software.

### VOR Recordings and Analysis

Adult C57BL/6 (C57BL/6JOlaHsd) mice were surgically prepared for experiments under general anaesthesia with isoflurane/O2, as previously described [72], [73]. After 5 days of recovery, mice were head-fixed to a metal bar with the body placed in a restraining device. The head of the mouse was placed in the centre of a turntable (diameter 60 cm) surrounded by a cylindrical screen (diameter 63 cm) with a random dotted pattern. An oil drive (Narishige, Tokyo, Japan) guided a double-barrel glass electrode (Septum Theta, WPI, FL, USA) into the brain. The double barrel had a 10 µm tip diameter, and was filled with 2M NaCl solution on the recording side and either saline or 5–10 ng (0.08–0.16 µl) of 135 µM nicotine solution labelled with Alcian Blue (dye) on the injection side. The left flocculus was located on the basis of Purkinje cell firing modulation recorded extracellularly during optokinetic stimulation (typically at 0.4 Hz with 8°/s peak velocity). Purkinje cells, identified by the occurrence of complex spikes, which responded optimally to horizontal, but not vertical, movements, were considered to indicate the VA zone. Upon localisation of the granule cell layer near the VA zone, air pressure was used to inject the nicotine solution (at ∼0.02–0.04 µl/min). Pre-training VOR was tested by rotating the table at 0.6 Hz through 5° in the dark, 15 min after completing the injection. Subsequently, mice were trained to decrease the gain of their VOR by means of five 10 min in-phase visual and vestibular stimulations (in both cases 5° at 0.6 Hz), while VOR was monitored in between and at the end of the stimulations. Eye movements were recorded with pupil tracking software (ISCAN, MA, USA), and analysed offline using custom-made Matlab routines. After the eye movement adaptation was recorded, mice were deeply anaesthetised with pentobarbital (200 mg/kg) and perfused transcardially with 4% paraformaldehyde. The brains were postfixed for 1 hour in 4% paraformaldehyde, embedded in gelatin (11%) and sectioned transversely at 40 µm using a freezing microtome, prepared for histology and analysed with a Leica DMR light microscope equipped with a DC 300 digital camera. To correlate the 3D injection sites with a map of the flocculus [73], digital prints were prepared of all consecutive sections from the caudal-most to the rostral-most part of the flocculus. Reference points were used to locate the injection in 2D map of the unfolded flocculus. Nicotine injections were included only if the dye covered at least 10% of the VA area. Linear regression revealed a positive correlation between dye-covered area and the post-training normalised gain with R = 0.60.

#### Statistical analysis

Data are reported as mean±SEM and the statistical significance of results was assessed. In case-control measurements and for comparisons between groups, a paired Student’s t-test was used.
